# Supplementary material for: Nutrition Transition in the Post-Economic Crisis of Greece: Assessing the Nutritional Gap of Food-Insecure Individuals. A Cross-Sectional Study
Source: Nutrients. 2019 Dec 2;11(12):2914. doi: 10.3390/nu11122914 (PMC6950031; doi:10.3390/nu11122914)
Supplement: Supplementary file 1 [file nutrients-11-02914-s001.pdf]

Supplementary Materials:

Table S1. Analysis of the adherence to the Greek Food-Bosed Dietary Guidelines per food group.

Food group	Recommendations	Serving Size	Recommended Grams per day	% meets the recommendations Cases Vs. Control
**Vegetables**	4 servings per day	150-200 gr	600 gr	0%—0%
**Legumes**	3 servings per week	150-200 gr	65 gr	33.5%—13.6%
**Fruits**	3 servings per day	120-200 gr	360 gr	0%—0%
**Cereal**	5-8 servings per day (3 servings of each category)	Cereal, Bread 30 gr Pasta/rice 70-90gr	90 gr 240 gr M.O. = 165 gr	41.7%—38.3%
**Dairy**	2 servings per day	Milk 250ml Yogurt 200 gr	400 gr	13.4%—19.1%
**Red meat**	Up to 1 serving per week	120-150 gr	17 gr	86.8%—93.6%
**White meat**	1-2 serving per week	120-150 gr	17 gr	81.8%—86.2%
**Fish and seafood**	2-3 servings per week	150 gr	43 gr	9.4%—15.4%
**Olive oil, olives & nuts**	4-5 servings per day	15ml, 1 handful	80 gr	45.3%63.3%

gr: grams.

Table S2. Analysis of the adherence to the WHO Eastern Mediterranean Region Food-Based Dietary Guidelines per food group.

Food group	Recommendations	Serving Size	Recommended Grams per day	% meets the recommendations Cases Vs. Control
**Vegetables**	5 servings per day	½ cup of cut-up raw or cooked vegetables, 1 cup raw leafy vegetable 90 gr	450 gr	**4.2%—16.1%**
**Legumes**	Included in Vegetable group	3 cups per week (1 cup = 150 gr)	64 gr	**33.5%—13.6%**
**Fruits**	4 servings per day	½ cup of fresh fruit, 1 medium fruit, ½ cup of fresh fruit juice 90 gr	360 gr	**0%—0%**
**Cereal**	180g per day	30 g (1 slice of bread, 1 cup of dry cereal, ½ cup cooked rice, pasta)	180 gr	**33.3%—30.1%**
**Dairy**	3 cups per day	1 cup of low-fat milk or yogurt, 45 g low-fat cheese	150 gr	**65.1%—62%**
**Red meat**	Meat and equivalents 160g per day	30 g	26 gr	**69.9%—89.2%**
**White meat**		30 g	52 gr	**81.8%—86.2%**
**Fish and seafood**		30 g	52 gr	**15.2%—31.4%**
**Eggs**		1egg = 44 gr	35 gr	**15.6%—12.8%**
**Olive oil, olives & nuts**	Oils: 6 tablespoons per day. Nuts are included in Meat and equivalents	1 tablespoon 15 g nuts	13.5 gr	**96.4%—98.2%**

gr: grams.
